# Beyond Religiosity: A Model to Explain Spirituality Among Middle-Aged and Older Adults

**DOI:** 10.1093/geroni/igaa057.1333

**Published:** 2020-12-16

**Authors:** Carlyn Vogel, Debra Dobbs, Brent Small

**Affiliations:** University of South Florida, Tampa, Florida, United States

## Abstract

Spirituality is difficult to define as researchers assign it different meanings and individuals’ perceptions can vary. For example, spirituality may connect to religiosity, while others consider religiosity a less significant part of spirituality. This study investigates factors outside of religiosity that are significantly associated with spirituality to inform the characteristics of the concept. Webster’s (2004) existential framework of spirituality was used to guide variable selection. The National Survey of Midlife in the United States wave three (MIDUS 3; 2013-2014; n = 2,594; Mage = 63.5, SD = 11, range = 39–92) was used to examine individuals’ reported levels of spirituality. Multinomial logistic regression was conducted to examine factors related to low and high levels of spirituality compared to a moderate level. Participants with low spirituality were more likely to be male, less likely to be mindful, mediate/chant, feel a strong connection to all life, to indicate that they cannot make sense of the world, and to be religious. Participants with high spirituality were more likely to be female, have at least some college experience, be mindful, meditate/chant, feel deep inner peace, have a sense of deep appreciation, think that a sense of purpose is important for a good life, and have a high level of religiosity. Framed by Webster’s conceptual model, the current study observed that religiosity is significantly associated with spirituality and that other mindfulness-based aspects are also present within this concept. Incorporating mindfulness with religious efforts will more accurately and holistically address spirituality.

